# OCH Ameliorates Bone Marrow Failure in Mice via Downregulation of T-Bet Expression

**DOI:** 10.1155/2014/928743

**Published:** 2014-08-28

**Authors:** Xiaohong Qiao, Xiaotian Xie, Wei Shi, Jinqing Tang, Yuexia Shao, Fuxing Li

**Affiliations:** Department of Paediatrics, Tongji Hospital, Tongji University, 389 Xincun Road, Shanghai 200065, China

## Abstract

The aim of this study is to evaluate the immune mechanism of OCH in the treatment of AA (also named bone marrow failure, BMF) induced in mice. OCH at a dose of 400 *μ*g/kg was injected intraperitoneally (I.P.) prior to the induction of BMF. Our study showed that the incidence of BMF was 100% in BMF group and 13% in OCH treatment group. Significant higher level of IL-4 and lower level of IFN-*γ* were observed in OCH group than that in BMF group (*P* < 0.05) as well as untreated group over BMF (*P* < 0.05). However, there was no significant difference between OCH and untreated group. Compared with untreated, the expression level of T-bet in OCH and BMF was all significantly higher. However, T-bet expression level was lower in OCH than in BMF. In addition, OCH treatment increased NKT cell fractions of bone marrow and the colonies of CFU-GM. In conclusion, treatment of OCH prior to the induction of BMF could prevent the incidence of BMF possibly through downregulating T-bet expression leading to the transition of immune response from Th1 to Th2, suggesting OCH might be a new therapeutic approach in the treatment of BMF or AA.

## 1. Introduction

Aplastic anemia (AA), also known as bone marrow failure (BMF), is a blood disorder, characterized by impaired hematopoiesis, leading to pancytopenia [1]. The pathogenesis of AA is very complicated and the impaired hematopoiesis from bone marrow was reported to be related with abnormal numbers and functions of T lymphocytes as well as dysregulation of cytokine secretion, suggesting that AA was an autoimmune disease, characterized by increased Th1 cells and downstream cytokines [2–4]. T-bet (T-box expressed in T cell), a member of T-box family discovered in 2000, is a Th1-specific transcription factor, responsible for Th1 cell differentiation [5]. Recent studies demonstrated that the expression level of T-bet was upregulated in AA, suggesting its involvement in the pathogenesis of AA [6, 7].

OCH is a sphingosine truncated derivative of alpha-galactosylceramide (*α*-GC) and can stimulate NKT cells to selectively produce Th2 cytokines, leading to the transition of immune response from Th1 to Th2 [8]. Previous studies demonstrated different cytokines secretion from NKT cell by different stimulus, with IFN-*γ* predominant by *α*-GC stimulation and IL-4 by OCH [9]. Given the critical role in the Th2 immune response via NKT activation, OCH has been under extensive studies as a therapeutic in the treatment of many Th1-related autoimmune diseases, such as experimental encephalomyelitis (EAE), type I diabetes, and collagen-induced arthritis (CIA) [10–13].

Abnormal activation of Th1 cells was thought to be the main cause of AA and OCH which was potentially helpful in the treatment of Th1-related disorders; therefore, we speculated that OCH might be used for AA treatment. Our previous studies showed administration of OCH could increase the overall survival in mice with BMF [14]. However, the exact mechanism underlying the effect of OCH on bone marrow remains poorly understood. The aim of this study was to investigate the effect of OCH on the expression level of T-bet, the percentage of NKT cells, the colonies of CFU-GM, secretion of IL-4 and IFN-*γ*, and the pathology of spleen and bone marrow in mice with immune-induced BMF. Our results showed that single dose OCH prior to the induction of BMF could ameliorate BMF possibly via downregulating T-bet, leading to the transition of immune response from Th1 to Th2.

## 2. Materials and Methods

### 2.1. Induction of BMF in Mice

Inbred male C57BL/6 (B6) and female BALB/cBy (BALB) mice were purchased from the Shanghai Animal Laboratory of Chinese Academy of Sciences. Hybrid CByB6F1 mice were housed at animal facilities of the Animal Laboratory of Tongji Hospital affiliated to Tongji University. Animals were provided with standard animal care, free access to diet and water. Males and females were selected from 6 to 16 weeks of age.

BMF was induced in mice as previously described [14]. Briefly, CByB6F1 mice received total body irradiation with a sublethal dose of 5.5 Gy (^137^cesium, ^137^cesium source was provided by the Department of radiology, Fudan University, Shanghai, China) followed by the infusion of lymphocytes isolated from B6 mice to induce bone marrow failure. Tail vein blood and bone marrow were collected on day 14 after lymphocytes infusion and complete blood count and bone marrow biopsy were performed to evaluate the success of induction of bone marrow failure in mice.

### 2.2. Experimental Groups Design

The mice were randomly divided into five groups as follows: untreated group (CByB6F1 mice); irradiation group (CByB6F1 mice received ^137^cesium); BMF group; OCH group (OCH was kindly provided by the National Institutes of Health Tetramer Facility; OCH was dissolved in PBS with 10% dimethyl sulfoxide (DMSO) and 400 *μ*g/kg OCH administration prior to the induction of BMF); control group (PBS with 10% DMSO administration).

### 2.3. Complete Blood Count

Lateral tail vein blood was drawn on d14 (day 14) and d60 after BMF induction into a tube with EDTA as anticoagulant. The numbers of white blood cell (WBC), red blood cell (RBC), hemoglobin (HB), and platelet (PLT) were measured by automated blood cell counter.

### 2.4. Measurement of Level of IFN-*γ* and IL-4 in Serum by ELISA

Orbital sinus blood was drawn on d30 and 60 after induction of BMF and was left at room temperature for 24 h followed by centrifuging at 3000 rpm for 5 min. The supernatant was isolated to obtain serum. The level of IFN-*γ* and IL-4 in serum was measured by ELISA kit according to the manufacturer's instructions (Bender, Vienna, Austria). Absorbance was read at 450 nm. All samples were analyzed in triplicate.

### 2.5. Assessment of Pathology of Bone Marrow and Spleen

Pathology of bone marrow and spleen was evaluated by hematoxylin and eosin (H&E) staining as previously described [14]. Briefly, on d14 and d60 after BMF induction, mice were sacrificed. Bone marrow and spleen were collected, fixed, embodied, sectioned, stained with H&E, and examined by using an Olympus microscope. Photographic images of bone marrow and spleen morphology were captured by using a digital camera.

### 2.6. CFU Assays

Methylcellulose semisolid culture medium was obtained as previously described [14] Briefly, untreated mice, BMF mice, and OCH treated mice were euthanized on day 60 after LN cells infusion. BM cells were extracted aseptically from one-side thigh-bone of mouse. BMMNCs were isolated through density gradient centrifugation by using mouse lymphocyte separation medium (Haoyang, Tianjin, China), washed twice with PBS, and then resuspended in RPMI-1640 (Gibco, Rockville, MD, USA). BMMNCs were planted into 24-well plates (Costar, Corning, USA) containing the relevant CFU-GM semisolid culture medium. For colony forming cell assays, colonies > 50 cells were counted under an inverted microscope on day 7.

### 2.7. Assessment of NKT Cell Percentages by Flow Cytometry

NKT cell percentages were analyzed in the BMMNCs from BMF group, untreated group and OCH treated group on day 60 after LN cells infusion by using flow cytometry according to a previous study [14]. Briefly, 1 × 10^6^ lymphoid cells were allocated for staining. The cells were washed with wash buffer (0.1% sodium azide, 0.1% bovine serum albumin in PBS) and resuspended in the 50 *μ*L of the same buffer. One 0.5 *μ*L test of fluorescently-labeled tetramers was added for staining and incubated on ice for 30 minutes in the dark and then washed with wash buffer. Aliquot of optimized FITC-labeled anti-mouse CD3 antibody was added for each staining and then incubated on ice for 30 minutes in the dark. The cells were washed twice with wash buffer and stored in fixative solution (1% fetal calf serum, 2.5% formaldehyde in PBS) in the dark until analysis. NKT cells were characterized as the CD3^+^CD1d/*α*-GC tetramers ^+^ population.

### 2.8. Measurement of T-Bet Expression Level in Spleen by Immunohistochemical Staining

Mice spleen pathology section was used to measure T-bet expression level in spleen by a commercial immunohistochemical staining kit according to manufacturer's instructions (anti-T-bet monoclonal antibody was from Santa Cruz Biotechnology lnc., USA). The positive cells were defined as tawny granular in cytoplasm and/or nucleus. Five nonrepetitive fields were randomly selected from positive cell region under 10 × 40 magnification and the numbers of positive cells in each field were used to quantify the expression level of T-bet. The percentage of positive cells was defined as (numbers of positive cells/total cells) × 100%.

### 2.9. Statistical Analysis

All the data were expressed as mean ± SD. For comparison of blood count, mice weight, NKT cell percentages, T-bet expression level, colonies of CFU, and serum level of IL-4 and IFN-*γ* among different groups, data was assessed by one-way ANOVA using SPSS20.0 software. Kaplan-Meier estimator was used for estimating the cumulative survival probability after treatment. Statistical significance was defined as the *P* value less than 0.05 (*P* < 0.05).

## 3. Results

### 3.1. BMF Induction

**Figure 1:**
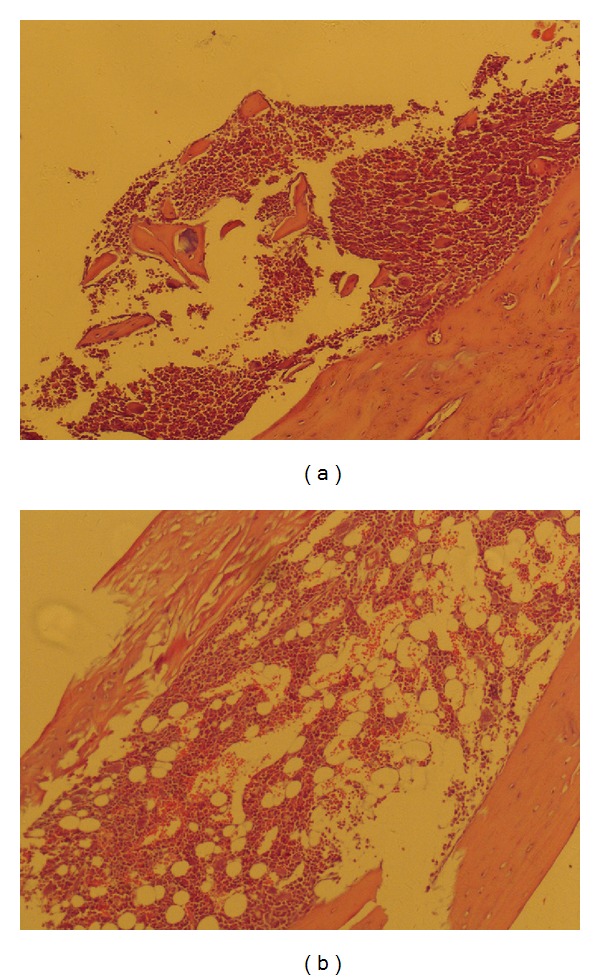
Pathology of bone marrow in mice with untreated (a) or BMF (b). On d14 after BMF induction, mice were sacrificed. Bone marrow were collected, fixed, embodied, sectioned, stained with H&E, and examined by using an Olympus microscope.

On d14 after BMF induction, complete blood count including WBC, RBC, HB, and PLT was dramatically decreased (Table 1) and bone marrow dysplasia with increased proportion of nonhematopoietic cells (Figure 1) were observed in all the treated mice, indicating the success of BMF induction. Compared to untreated mice, bone marrow dysplasia, reduced hematopoietic tissue region, increased fatty tissue, and decreased numbers of megakaryocytes and hematopoietic cells were seen in BMF mice (Figure 1). In addition, sinus congestion, bleeding, and edema were also found in bone marrow.

### 3.2. Mice Weight

There was no significant difference in each group before treatment, regarding the mice weight. Mice weight was increased on d5 in all treatment groups, including the untreated group (Table 2), compared to day 0. However, the increasing extent was less in all treatment groups than untreated as significantly lower weight was observed on d5 (*P* < 0.01). Compared to BMF group (19.9 ± 1.0 g), mice from irradiation (20.0 ± 1.0 g) or OCH (20.0 ± 1.0 g) group had significantly higher weight (*P* < 0.01), but lower than that of untreated group (21.8 ± 1.0 g). Relatively lower weight of mice from irradiation (19.9 ± 0.9 g) or OCH group (19.6 ± 0.9 g) and obviously reduced weight of mice from BMF (14.9 ± 0.7 g) or control group (15.0 ± 0.7 g) were found on d10 compared to that on d5. Mice weight from treatment groups on d10 was still significantly lower than that of untreated group (21.7 ± 0.8 g) (*P* < 0.01).

### 3.3. Mortality, Incidence of BMF, and Overall Survival

**Figure 2:**
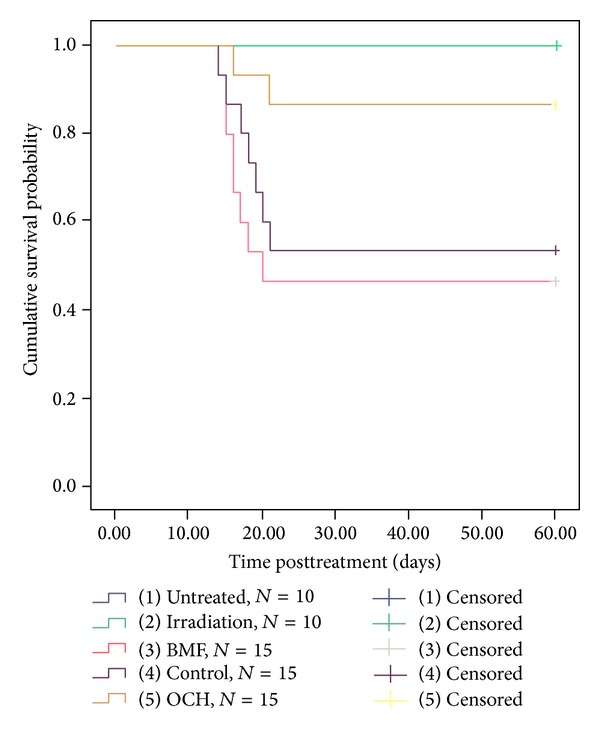
Kaplan-Meier analysis of overall survival in mice from different groups.

During 60 days of follow-up, no mortality was observed in the mice from untreated and irradiation groups (Table 3). 8 mice died in BMF group and 7 died in control group. BMF occurred in all the remaining mice in BMF and control groups (Table 3). However, only 2 died in OCH group without occurrence of BMF, suggesting the protective effect of OCH in the development of BMF. Kaplan-Meier analysis of overall survival (Figure 2) showed that there was no significant difference between untreated and OCH group (*P* > 0.05) as well as between control and BMF group (*P* > 0.05). However, a significant lower overall survival was seen in BMF group when compared to OCH group (*P* < 0.05), same to control group compared with untreated (*P* < 0.05).

### 3.4. Complete Blood Cell Count after Treatment

On d14 posttreatment, numbers of WBC, RBC, Hb, and PLT (Table 4) in BMF and OCH groups were significantly lower compared to that in untreated group (*P* < 0.01). However, significant lower numbers of complete blood cell were observed in BMF compared to OCH group (*P* < 0.01). No statistical difference was seen between irradiation versus untreated group and control versus BMF group (*P* > 0.05), except lower number of WBC found in irradiation than in untreated group (*P* < 0.01).

On d60 after treatment, complete blood cell count (Table 5) was normalized in OCH and irradiation groups and remained still significantly lower in BMF and control groups compared to untreated group (*P* < 0.05).

### 3.5. Serum Level of IFN-*γ* and IL-4

Serum was isolated from orbital sinus blood taken from untreated, BMF, and OCH groups of mice on d30 and d60 posttreatment and used to measure level of IFN-*γ* and IL-4. Compared to untreated and OCH groups, higher level of IFN-*γ* and lower level of IL-4 were found in BMF group. However, no significant difference was observed between untreated and OCH groups (Table 6).

### 3.6. Pathology of Bone Marrow and Spleen

**Figure 3:**
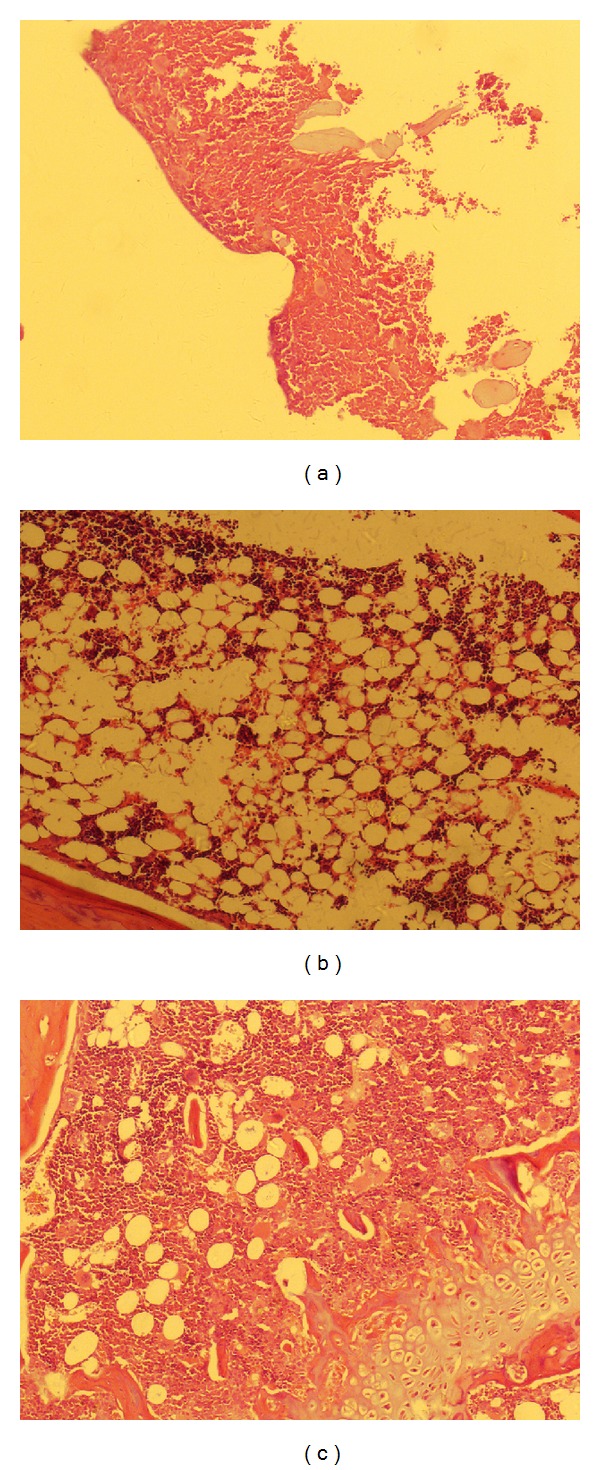
Pathology evaluation of bone marrow on d60 in mice with untreated (a), BMF (b), or OCH (c). On d60 after BMF induction, mice were sacrificed. Bone marrow was collected, fixed, embodied, sectioned, stained with H&E, and examined by using an Olympus microscope.

**Figure 4:**
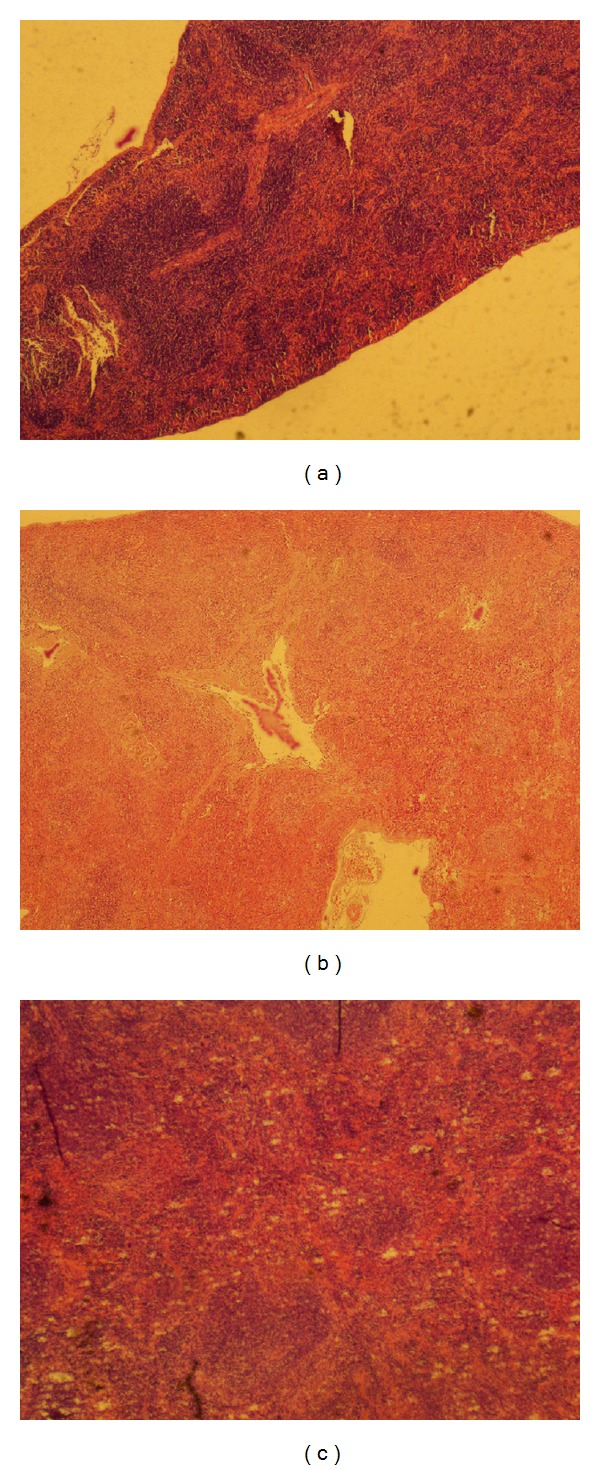
Pathology evaluation of spleen on d60 in mice with untreated (a), BMF (b), or OCH (c). On d60 after BMF induction, mice were sacrificed. Spleens were collected, fixed, embodied, sectioned, stained with H&E, and examined by using an Olympus microscope.

Bone marrow and spleen were collected to measure pathology changes on d60 posttreatment. Compared to untreated mice, similar pathology changes of bone marrow to that observed on d14 after induction described in a previous section (BMF induction) were seen in BMF group (Figure 3). More hematopoietic cells and less nonhematopoietic cells were found in OCH group. Regarding the spleen pathology, compared to untreated mice (Figure 4(a)), the following changes were observed in BMF mice: loose structure of spleen tissues, indistinct spleen lymph follicle structure, reduced acini lienalis, obscure germinal center, dilation of splenic sinusoid, and thickening and hyalinization of splenic arteriole wall in some areas (Figure 4(b)). However, all the pathological changes occurred in BMF mice were improved in OCH group (Figure 4(c)), indicating that OCH treatment could ameliorate BMF associated with the damage to bone marrow and spleen.

### 3.7. Increased Numbers of Mononuclear Cells and CFU by OCH

On d60 after treatment, expanded incubation of CFU colony in the CFU-GM semisolid culture medium was conducted for 7 days for the untreated, BMF, and OCH groups of mice. Cells with more than 50 colonies were counted. The numbers of mononuclear cells in BM and CFU in the BMF mice were much lower than that in untreated mice and OCH mice (*P* < 0.01) (Table 7). No difference in mononuclear cell numbers and CFU was found between untreated mice and the OCH-treated mice.

### 3.8. Increased NKT Cell Fraction of BMMNCs by OCH Treatment

**Figure 5:**
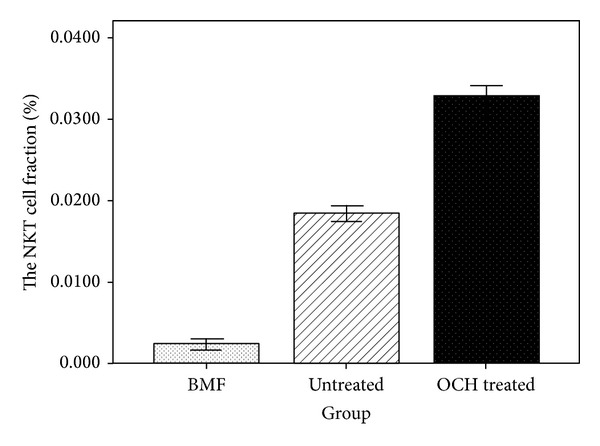
A fraction of NKT cells in BMMNCs. On d60 after BMF induction, NKT cell percentages of BMMNCs from BMF, untreated, and OCH groups were analyzed. Compared with the untreated group, there were fewer NKT cells of BMMNCs in BMF group (*P* < 0.01, by ANOVA, *n* = 7). NKT cell population was increased in OCH group. The NKT cell percentage of BMMNCs was found with statistical differences between the OCH group and BMF group (*P* < 0.01, by ANOVA, *n* = 7).

Compared with the untreated mice, fewer NKT cell fraction of BMMNCs in BMF mice (0.24 ± 0.03 versus 1.84 ± 0.05%, *P* < 0.01, *n* = 7) were found. Proportional NKT cells of BMMNCs were higher in the OCH group mice (3.28 ± 0.07%, *n* = 7) than those in BMF mice. (*P* < 0.01) (Figure 5). These data revealed that NKT cell population of BMMNCs in BMF mice was decreased, whereas OCH increased NKT cell population.

### 3.9. T-Bet Expression Level in Spleen

**Figure 6:**
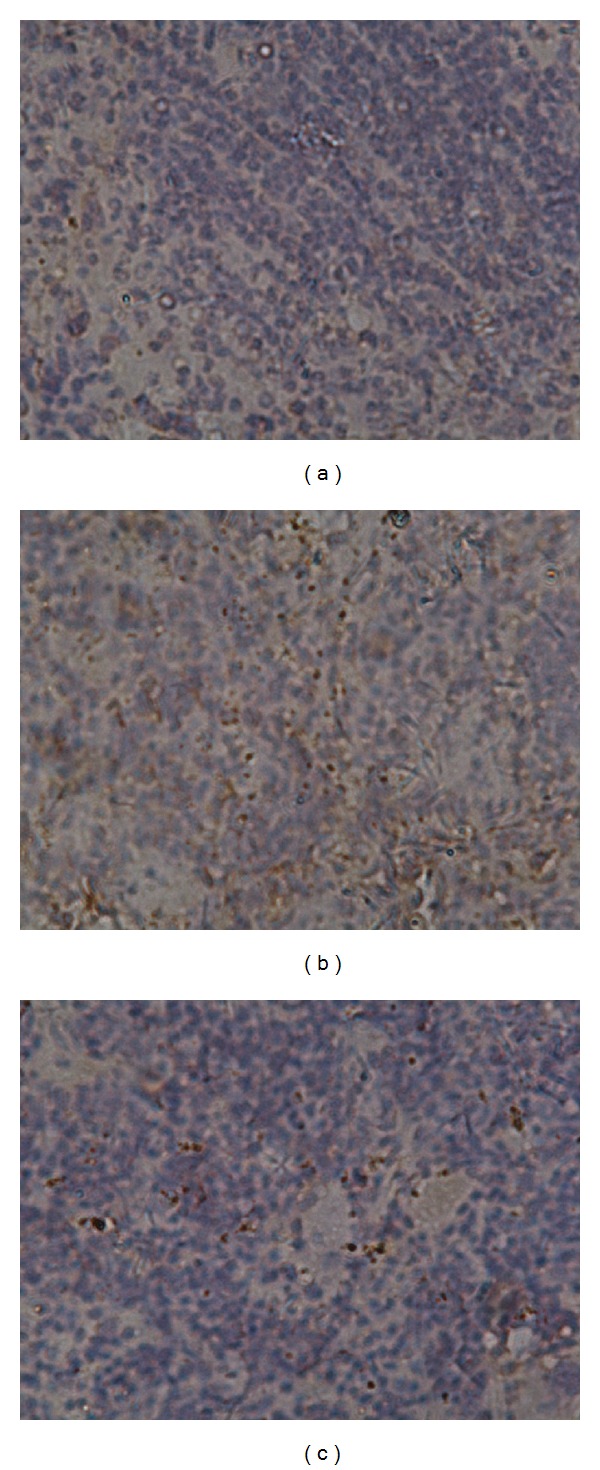
T-bet expression in spleen on d60 in mice with untreated (a), BMF (b), or OCH (c). Mice spleen pathology section was used to measure T-bet expression level in spleen by immunohistochemical staining. The positive cells were defined as tawny granular in cytoplasm and/or nucleus.

Immunohistochemical staining was used to determine the protein expression level of T-bet in spleen. As seen in Figure 6, significantly higher expression level of T-bet was seen in BMF group (8.25 ± 0.05%) than that in either untreated (0.78 ± 0.05%, *P* < 0.01) or OCH group (3.25 ± 0.06%, *P* < 0.01), suggesting the role of T-bet in the development of BMF as well as the downregulation of T-bet expression by OCH. However, T-bet level in OCH group was still higher than in untreated group (*P* < 0.01).

## 4. Discussion

AA is a blood disease, characterized by reduced hematopoiesis, resulting in a deficiency of all three blood cell types, red blood cells, white blood cells, and platelets [15]. AA has been known as an autoimmune disease, characterized by Th-1-mediated abnormal immune response. There are a growing numbers of studies demonstrating the association of abnormal numbers and function of NKT cells with many autoimmune diseases, such as diabetes, multiple sclerosis, and rheumatoid arthritis [8, 16]. CD1d is known to activate NKT cells as an antigen presenting molecule [17] and CD1d-deficient mice displayed more frequent and severe skin disease as well as increased local inflammation with infiltration of lymphocytes and dendritic cells in MRL-lpr/lpr mice [18]. Previous studies demonstrated that activated NKT cells could inhibit autoimmune diabetes [19] or EAE [10] in mice, suggesting the negative immune-regulatory role of NKT cells as further supported by previous studies showing reduced numbers of NKT cells in patients with AA [20, 21]. However, the exact role of NKT cells in AA remains poorly understood. In this study, we showed reduced numbers of NKT cells in mice with BMF and increased after administration of OCH, suggesting the involvement of NKT cells in the pathogenesis of AA as an immunoregulatory factor of Th1/Th2.

Single administration of OCH could prevent EAE, characterized by Th1-mediated autoimmune disease, via inducing Th2 bias of NKT cells to produce IL-4 [10]. Our studies revealed that single administration of OCH could prevent BMF in mice, possibly through activation of NKT cells as previous studies reported that no preventative effect of OCH was observed in NKT cell deficient mice CIA [22]. Given the important role in the autoimmune regulation, stimulating NKT cell activation by OCH could be beneficial in the treatment of many autoimmune diseases, including AA.

The pathogenesis of AA is very complicated, with lots of factors involved leading to the damage to bone marrow and subsequent impaired hematopoiesis. Recent studies on it are mainly focused on the abnormal regulation of autoimmunity, especially subsets and dysfunction of T lymphocytes [23–25]. Based on the secreted cytokines and functions, there are two mainly subsets of T cells, Th1 and Th2, which produce IFN-*γ*, IL-2, TNF-*α*, and IL-4, IL-5, IL-6, IL-10, and IL-13, respectively [26]. Consistent with AA as a Th1-mediated autoimmune disease, mice with BMF displayed higher level of IFN-*γ* and lower level of IL-4. T-bet is a recently identified transcription factor belonging to T-box family [5]. As a Th1-specific transcription factor, its expression determines the differentiation of resting CD4+T cells to Th1 cells via stimulating IFN-*γ* expression [27]. In addition, T-bet could induce and maintain the expression level of IL-12R*β*2 and reverse Th2 cells into Th1 with the production of IFN-*γ* which inhibits secretion of Th2 cytokines, such as IL-4, IL-5, and IL-13 [28]. Our studies demonstrated higher expression level of T-bet in spleen in mice with BMF compared to untreated, as well as the subsequent higher level of IFN-*γ*, suggesting the involvement of T-bet in the development of AA. However, T-bet and IFN-*γ* level were lower in OCH group than in BMF, implying the protective role of OCH in the development of BMF possibly through downregulating T-bet expression. Meanwhile, less severe damage to bone marrow and spleen was observed in OCH group than in BMF, indicating the protective effect of OCH on bone marrow and spleen in BMF mice.

In conclusion, our studies showed that OCH, as a glycolipid ligand for NKT cells, could protect BMF in mice through downregulating T-bet expression, leading to reduced cytokine secretion of IFN-*γ*, increased secretion of IL-4 and numbers of CFU-GM as well as NKT fractions, ultimately resulting in the transition of immune response from Th1 to Th2. Therefore OCH might be as a new therapeutic strategy in the prophylaxis and treatment of AA.

## Figures and Tables

**Table 1:** Complete blood cell count in untreated and BMF group (*n* = 10).

Group	WBC (×10^9^/L)	RBC (×10^12^/L)	HB (g/L)	PLT (×10^9^/L)
Untreated	8.24 ± 0.12	9.1 ± 0.6	165 ± 8	940 ± 16
BMF	0.46 ± 0.13	3.1 ± 0.7	42 ± 8	86 ± 15
*F* value	0.207	0.415	0.03	0.075
*t* value	137.908	20.381	33.752	122.923
*P* value	0.000	0.000	0.000	0.000

**Table 2:** Mice weight in different groups (g).

Group	Sample number	d0	d5	d10
Untreated	10	18.0 ± 0.4	21.8 ± 1.0^▲^	21.7 ± 0.8^▲^
Irradiation	10	18.0 ± 0.4	20.0 ± 1.0^△▲^	19.9 ± 0.9^△▲^
BMF	15	18.0 ± 0.4	19.9 ± 1.0^△^	14.9 ± 0.7^△^
Control	15	18.0 ± 0.3	19.9 ± 0.9^△^	15.0 ± 0.7^△^
OCH	15	18.0 ± 0.4	20.0 ± 1.0^△▲^	19.6 ± 0.9^△▲^
Statistic		*F* = 0.06	*F* = 7.741	*F* = 146.234
*P* value		1	0.000	0.000

Compared with untreated, ^△^ *P* < 0.01. Compared with BMF, ^▲^ *P* < 0.01.

**Table 3:** Incidence of mortality and BMF in mice with different treatment.

Group	Sample number	Mortality	BMF
Untreated	10	0	0
Irradiation	10	0	0
BMF	15	8	7
Control	15	7	8
OCH	15	2	0

**Table 4:** Complete blood cell count on d14 after treatment.

Group	WBC (×10^9^/L)	RBC (×10^12^/L)	HB (g/L)	PLT (×10^9^/L)
Untreated (*n* = 10)	8.23 ± 0.11^▲^	9.3 ± 0.7^▲^	163 ± 9^▲^	940 ± 8^▲^
Irradiation (*n* = 10)	4.46 ± 0.11^△▲^	9.1 ± 0.7^▲^	160 ± 8^▲^	936 ± 9^▲^
BMF (*n* = 14)	0.44 ± 0.10^△^	3.1 ± 0.9^△^	43 ± 8^△^	87 ± 9^△^
Control (*n* = 14)	0.45 ± 0.09^△^	3.2 ± 0.7^△^	42 ± 8^△^	89 ± 9^△^
OCH (*n* = 15)	4.25 ± 0.10^△▲^	6.3 ± 0.8^△▲^	108 ± 7^△▲^	560 ± 8^△▲^
Statistic	*F* = 10531.525	*F* = 211.535	*F* = 670.289	*F* = 28505.728
*P* value	0.000	0.000	0.000	0.000

Compared with untreated, ^△^ *P* < 0.01. Compared with BMF, ^▲^ *P* < 0.01.

**Table 5:** Complete blood cell count on d60 after treatment.

Group	WBC (×10^9^/L)	RBC (×10^12^/L)	HB (g/L)	PLT (×10^9^/L)
Untreated (*n* = 10)	8.43 ± 0.08^▲^	9.3 ± 0.6^▲^	163 ± 8^▲^	880 ± 10^▲^
Irradiation (*n* = 10)	8.38 ± 0.07^▲^	9.1 ± 0.7^▲^	160 ± 7^▲^	878 ± 8^▲^
BMF (*n* = 7)	0.75 ± 0.10^△^	3.2 ± 0.6^△^	50 ± 9^△^	90 ± 8^△^
Control (*n* = 8)	0.74 ± 0.10^△^	3.5 ± 0.6^△^	51 ± 8^△^	94 ± 9^△^
OCH (*n* = 13)	8.37 ± 0.08^▲^	9.2 ± 0.6^▲^	161 ± 8^▲^	875 ± 10^▲^
Statistic	*F* = 14553.792	*F* = 236.290	*F* = 461.030	*F* = 20922.423
*P* value	0.000	0.000	0.000	0.000

Compared with untreated, ^△^ *P* < 0.01. Compared with BMF, ^▲^ *P* < 0.01.

**Table 6:** Serum level of IL-4 and IFN-*γ* (pg/mL, *n* = 7).

Group	IL-4		IFN-*γ*	
	d30	d60	d30	d60
Untreated	75 ± 4^▲^	73 ± 3^▲^	34 ± 3^▲^	34 ± 3^▲^
BMF	35 ± 3^△^	36 ± 3^△^	76 ± 4^△^	76 ± 3^△^
OCH	76 ± 4^▲^	74 ± 3^▲^	33 ± 3^▲^	34 ± 3^▲^

Compared with untreated, ^△^ *P* < 0.01. Compared with BMF, ^▲^ *P* < 0.01.

**Table 7:** The numbers of mononuclear cells and CFU.

Group	*N*	Mononuclear cells	CFU
		(×10^6^ a single thigh bone)	
Untreated	7	14 ± 0.6	50 ± 6
BMF	7	2 ± 0.1^△^	6 ± 1^△^
OCH	7	12 ± 0.6^▲^	46 ± 5^▲^

Compared with untreated, ^△^ *P* < 0.01. Compared with BMF, ^▲^ *P* < 0.01.

## Highlights

- OCH ameliorates immune-induced bone marrow failure in mice.
- Increased T-bet expression in mice model of bone marrow failure.
- Downregulation of T-bet expression by OCH.
